# Serum total bile acids within the normal range are inversely associated with inflammatory indices and Gensini score in patients with premature coronary artery disease

**DOI:** 10.3389/fimmu.2026.1790698

**Published:** 2026-04-16

**Authors:** Heze Fan, Wenyuan Li, Yuzhi Huang, Yirui Xu, Wenbo Yang, Erwei Hu, Wenjiao Zhang, Hao Yuan, Peiyao Zhang, Xueying Feng, Qi Kang, Chenbo Xu, Shumei Zhang, Ting Li, Zuyi Yuan, Juan Zhou

**Affiliations:** 1 Department of Cardiology, The First Affiliated Hospital of Xi’an Jiaotong University, Xi’an, Shaanxi, China; 2 Key Laboratory of Environment and Genes Related to Diseases, Ministry of Education, Xi’an, China

**Keywords:** Gensini score, immune, inflammation indices, premature coronary artery disease, total bile acid

## Abstract

**Background:**

Both serum bile acids and inflammation have been reported to be associated with coronary artery disease (CAD). However, whether serum total bile acid (TBA) can influence the extent of atherosclerosis in premature CAD (PCAD) patients by modulating immune-inflammation is unknown.

**Methods:**

A total of 631 patients with PCAD were enrolled from the First Affiliated Hospital of Xi’an Jiaotong University. Spearman correlation and logistic/linear regression were used to assess the associations between TBA and mediator/outcome variables. The mediating effect of immune-inflammation in the association of TBA with outcomes was further evaluated.

**Results:**

TBA was associated with a decreased risk of first myocardial infarction (MI) and a reduced degree of coronary stenosis in PCAD patients. Moreover, the inverse association between TBA and immune-inflammation was observed. Multivariate linear regression showed that for each unit increase in TBA, neutrophil (NEU) decreased by 0.27×10^9/L (β = -0.27, 95% CI: -0.37, -0.16), systemic inflammation response index (SIRI) decreased by 0.12 (β = -0.12, 95% CI: -0.17, -0.06), systemic immune-inflammation index (SII) decreased by 55.25 (β = -55.25, 95% CI: -85.78, -24.72), and C-reactive protein (CRP) decreased by 0.13 mg/L (β = -0.13, 95% CI: -0.25, -0.02). More importantly, the mediation analysis indicated that NEU, CRP, SII, and SIRI statistically explained the association between the TBA and outcomes.

**Conclusion:**

TBA is associated with reduced coronary lesion severity, an association that may be linked to chronic inflammation.

## 1 Introduction

Coronary artery disease (CAD) is the leading cause of death in patients with cardiovascular disease and is recognized as a major threat to human health worldwide. Although CAD is more common in the elderly, the risk of it has been trending younger in recent years (1). Premature coronary artery disease (PCAD) belongs to a special subgroup of CAD, which refers to the occurrence of CAD in men younger than 55 years of age and in women younger than 65 years of age. Because of the early age of onset and relatively longer life expectancy of PCAD patients, more than half of PCAD patients experience substantial progression of coronary atherosclerosis within 10 years, and one-fifth of patients die prematurely (2). Therefore, regular monitoring of PCAD patients to prevent progression of coronary artery disease is an important way to improve their prognosis. However, few studies have explored novel biomarkers in the PCAD population to enable risk assessment.

Bile acids are cholesterol metabolites synthesized in the liver and undergo enterohepatic circulation, playing a key role in cholesterol homeostasis (3). Animal studies have shown that increased bile acid excretion in the absence of hepatobiliary disorders counteracts the cholesterol load caused by a high-fat diet and delays the onset of atherosclerotic lesions (4, 5). Madeleine Alder et al. reported that the addition of bile acid sequestrants to statin drugs resulted in a further reduction in low-density lipoprotein cholesterol (LDL) levels (6). In addition, bile acid excretion is lower in patients with CAD than in those without CAD, suggesting that an imbalance in bile acid metabolism may play an important role in the development of CAD (7, 8).

Total bile acids (TBA) refers to the sum of all different types of bile acids in the blood. Clinically, the serum TBA level is a sensitive indicator of the liver’s synthetic, uptake, and excretory functions. Serum TBA has also been found to be strongly associated with the development of CAD. Serum TBA levels were significantly reduced in patients with CAD and myocardial infarction (9). Ting-Ting Liu et al. further demonstrated the protective effect of serum TBA on the prognosis of patients with acute coronary syndromes and the extent of coronary artery disease (10). Although serum TBA levels have been associated with CAD, no study has investigated serum TBA levels in patients with PCAD. The mechanism by which serum TBA affects the progression of CAD remains unclear.

Bile acids exert immunomodulatory effects primarily through the activation of Takeda G-protein-coupled receptor 5 (TGR5) and Farnesoid X Receptor (FXR). These two receptors are widely expressed on the surface of immune cells such as macrophages and monocytes, and their activation effectively inhibits the production of pro-inflammatory cytokines while promoting the formation of anti-inflammatory phenotypes (11–14). The signaling pathways mediated by TGR5 and FXR constitute critical nodes in the regulatory network connecting bile acid metabolism and immune-inflammation, suggesting that bile acids may influence the initiation and progression of cardiovascular diseases by modulating inflammatory processes. A recent experimental study has suggested that TBA may attenuate inflammatory response in patients with myocardial infarction (MI) (15).

Considering the role of immune-inflammation as a bridge between TBA and CAD, we made a first attempt to use mediating effects to analyze whether serum TBA might be inversely associated with the extent of coronary artery disease and the risk of first infarction in patients with PCAD by attenuating chronic inflammation.

## 2 Methods

### 2.1 Data sources and study population

We collected PCAD patients who underwent coronary angiography at the First Affiliated Hospital of Xi’an Jiaotong University between January 2018 and March 2019. The inclusion criteria of the study population were as follows: 1) Men younger than 55 years, women younger than 65 years, and who underwent coronary angiography (16, 17); 2) Confirmed diagnosis of CAD. The percentage of luminal stenosis was assessed by two or more experienced interventional cardiologists at our institution. CAD was diagnosed if there was a 50% or more lumen diameter stenosis in at least one major coronary artery.

The exclusion criteria for the study population were as follows: 1) autoimmune diseases; 2) Acquired Immune Deficiency Syndrome; 3) hyperthyroidism; 4) acute and chronic inflammatory diseases: such as acute and chronic gastritis, acute and chronic cholecystitis, acute and chronic bronchitis, etc; 5) Malignant tumors; 6) previous percutaneous coronary intervention; 7) Previous MI and stroke; 8) Missing TBA data and higher than the clinical reference value (10 µmol/L); 9) Missing data on important covariates; 10) Severe hepatic and renal dysfunction (creatinine >133 µmol/L and alanine aminotransferase >120 U/L); 11) dilated cardiomyopathy. Ultimately, 631 PCAD patients were included.

### 2.2 Assessment of atherosclerosis

MI: The diagnosis of MI requires the fulfillment of at least two of the following criteria, 1) symptoms of typical chest pain (pain in the precordial area for more than 30 minutes; 2) significant elevation of cardiac markers in serum, preferably high-sensitivity cardiac troponin, with at least one value above the 99th percentile of the upper limit of reference; 3) an electrocardiogram suggestive of MI; 4) loss of viable myocardium or new regional ventricular wall imaging evidence of motion abnormalities.

Chronic total occlusion (CTO): a coronary lesion with total occlusion of the coronary arteries, grade 0 forward TIMI flow, and occlusion for more than 3 months.

Gensini score: The base score was first determined by the degree of coronary artery stenosis, and then the corresponding coefficients were assigned to different coronary branches (18). The sum of the scores of each lesion vessel is the total score of the degree of coronary artery stenosis.

Severe coronary lesion (SCL): this study defined a Gensini score higher than 70% of the study population as SCL, i.e., Gensini score >61.

### 2.3 Measurements

Study subjects were admitted to the hospital, where clinicians took medical histories, including age, gender, smoking history, and past medical history. Blood samples were collected from all patients prior to coronary angiography. For patients with acute MI requiring emergency percutaneous coronary intervention, samples were obtained upon hospital admission prior to the procedure, while clinically stable patients underwent blood collection after an overnight fast. Serum fasting TBA was measured using the bile acid test kit (Pureauto S TBA) and an enzyme cycle assay. Indicators used to assess the inflammatory status included C-reactive protein (CRP), neutrophil (NEU), systemic inflammatory response index (SIRI), and systemic immune-inflammation index (SII). All indicators were analyzed by experts from the Department of Laboratory Medicine of the First Affiliated Hospital of Xi’an Jiaotong University using standard testing techniques. The tests were mainly performed by an automated hematology analyzer (Sysmex 2100, Japan) using whole blood. SIRI=NEU count × monocytes count/lymphocyte count (19). SII= platelet count × NEU count/lymphocyte count (19). All cell counts were expressed as ×10^9/L.

### 2.4 Statistical analysis

R software (version 4.1) was used to perform all statistical analyses. Means ± standard deviations indicate continuous variables, whereas frequencies or percentages indicate categorical variables. Based on the nature of the data, chi-square tests, ANOVA, or Kruskal-Wallis tests were performed to determine differences between participants across TBA tertiles. Spearman correlation analyses were performed to assess the correlation of TBA with other variables. Univariate and multivariate logistic regression models to assess the correlation of the exposure variable (TBA) with dichotomous outcome variables (MI, CTO, SCL). Univariate and multivariate linear regression models were used to assess the association between TBA and continuous outcome variables (Gensini score and inflammatory indicators). In addition, we also used restricted cubic spline (RCS) curves to visualize the association between exposure and outcome. Mediation analyses were performed using the “mediation” package of the R software. Each mediator was analyzed in separate single-mediator models, and the proportions mediated are not additive due to correlations/overlapping information among the mediators. Within the mediation analysis framework, linear regression models with an identity link function were applied to the continuous outcome (Gensini score), and logistic regression models with a logit link function were applied to binary outcomes (MI, CTO, and SCL). All mediation analyses were conducted using 5,000 bootstrap resamples (sims = 5000). All adjusted models, as well as the mediation analysis, were adjusted for the same covariates. The included covariates are: age, sex, hypertension, diabetes, smoking status, triglycerides, high-density lipoprotein, low-density lipoprotein, alanine aminotransferase, creatinine, HbA1c, fibrinogen, and family history of CAD.

TBA was the exposure variable. NEU, SII, SIRI, and CRP were the mediating variables. MI, CTO, SCL, and Gensini scores were the outcome variables. The existence of mediating effects requires the following conditions to be fulfilled: 1) Statistical significance must exist between the exposure variables and the inflammatory factors. 2) Statistical significance must exist between the inflammatory factors and the outcome variables. Therefore, if we analyzed the mediating effect only for the inflammatory factors that met the above conditions.

## 3 Results

### 3.1 Baseline characteristic

As shown in **Table 1**, a total of 631 PCAD patients were included, with a mean age of 51 years, and 60.86% of the participants were male. Patients were categorized into three groups by TBA tertiles, and we found that PCAD patients with lower TBA levels were more likely to be male and younger. Both alanine aminotransferase (ALT) and LDL were higher in PCAD patients with lower TBA. In addition, inflammatory markers—including NEU, monocytes, white blood cell (WBC), SIRI, and CRP—were significantly higher in PCAD patients with low TBA levels. More importantly, PCAD patients with lower TBA levels had an increased prevalence of MI, SCL, and CTO, as well as increased Gensini scores, compared with those with higher TBA.

**Table 1:** Baseline characteristics.

Variables	Total population (n= 631)	T1 (n= 216)	T2 (n= 206)	T3 (n= 209)	*P*-value
Age, (years)	51.13 (7.64)	49.67 (7.32)	52.06 (7.99)	51.71 (7.43)	<0.001
Sex, male, n (%)	384 (60.86%)	146 (67.59%)	115 (55.83%)	123 (58.85%)	0.036
Smoking, n (%)	296 (46.91%)	112 (51.85%)	90 (43.69%)	94 (44.98%)	0.193
Diabetes, n (%)	141 (22.35%)	41 (18.98%)	51 (24.76%)	49 (23.44%)	0.325
Hypertension, n (%)	298 (47.23%)	96 (44.44%)	103 (50.00%)	99 (47.37%)	0.520
Family history of CAD, (%)	134 (21.24%)	51 (23.61%)	45 (21.84%)	38 (18.18%)	0.379
Creatinine, µmol/L	59.04 (16.11)	60.77 (16.48)	58.67 (16.07)	57.63 (15.66)	0.101
ALT, U/L	33.66 (23.01)	39.91 (26.31)	31.78 (22.22)	29.05 (18.34)	<0.001
Triglycerides, mmol/L	1.83 (1.22)	1.78 (1.10)	1.78 (1.04)	1.92 (1.46)	0.991
HDL, mmol/L	0.99 (0.24)	0.98 (0.23)	1.00 (0.24)	0.99 (0.25)	0.742
LDL, mmol/L	2.41 (0.91)	2.55 (0.98)	2.43 (0.94)	2.25 (0.77)	0.004
HbA1c, %	6.00 (1.22)	5.93 (1.17)	6.11 (1.35)	5.98 (1.12)	0.185
Fibrinogen, g/L	3.02 (0.94)	3.03 (0.97)	3.05 (0.92)	2.98 (0.92)	0.669
NEU, 10^9/L	5.78 (3.15)	6.98 (3.39)	5.36 (3.14)	4.94 (2.46)	<0.001
MONO, 10^9/L	0.38 (0.18)	0.42 (0.21)	0.37 (0.16)	0.35 (0.14)	<0.001
WBC, 10^9/L	8.00 (3.32)	9.19 (3.54)	7.58 (3.33)	7.19 (2.69)	<0.001
SIRI	1.59 (1.65)	2.15 (1.85)	1.40 (1.56)	1.22 (1.35)	<0.001
SII	863.04 (851.97)	1,098.43 (883.38)	800.95 (991.42)	680.97 (573.14)	<0.001
CRP, mg/L	2.94 (3.26)	3.52 (3.62)	2.83 (3.05)	2.43 (2.97)	0.007
MI, (%)	218 (34.55%)	121 (56.02%)	58 (28.16%)	39 (18.66%)	<0.001
Gensini score	48.54 (38.80)	58.50 (42.34)	45.26 (36.10)	41.48 (35.48)	<0.001
SCL, (%)	189 (29.95%)	83 (38.43%)	58 (28.16%)	48 (22.97%)	0.002
CTO, (%)	145 (22.98%)	83 (38.43%)	38 (18.45%)	24 (11.48%)	<0.001

ALT, aminotransferase; CRP, C-reactive protein; CTO, Chronic total occlusion; HDL, high-density lipoprotein cholesterol; LDL, low-density lipoprotein cholesterol; MI, myocardial infarction; NEU, Neutrophil; SCL, Severe coronary lesion; SIRI, systemic inflammatory response index; SII, systemic immune-inflammation index; WBC, white blood cell.

**Figure 1:**
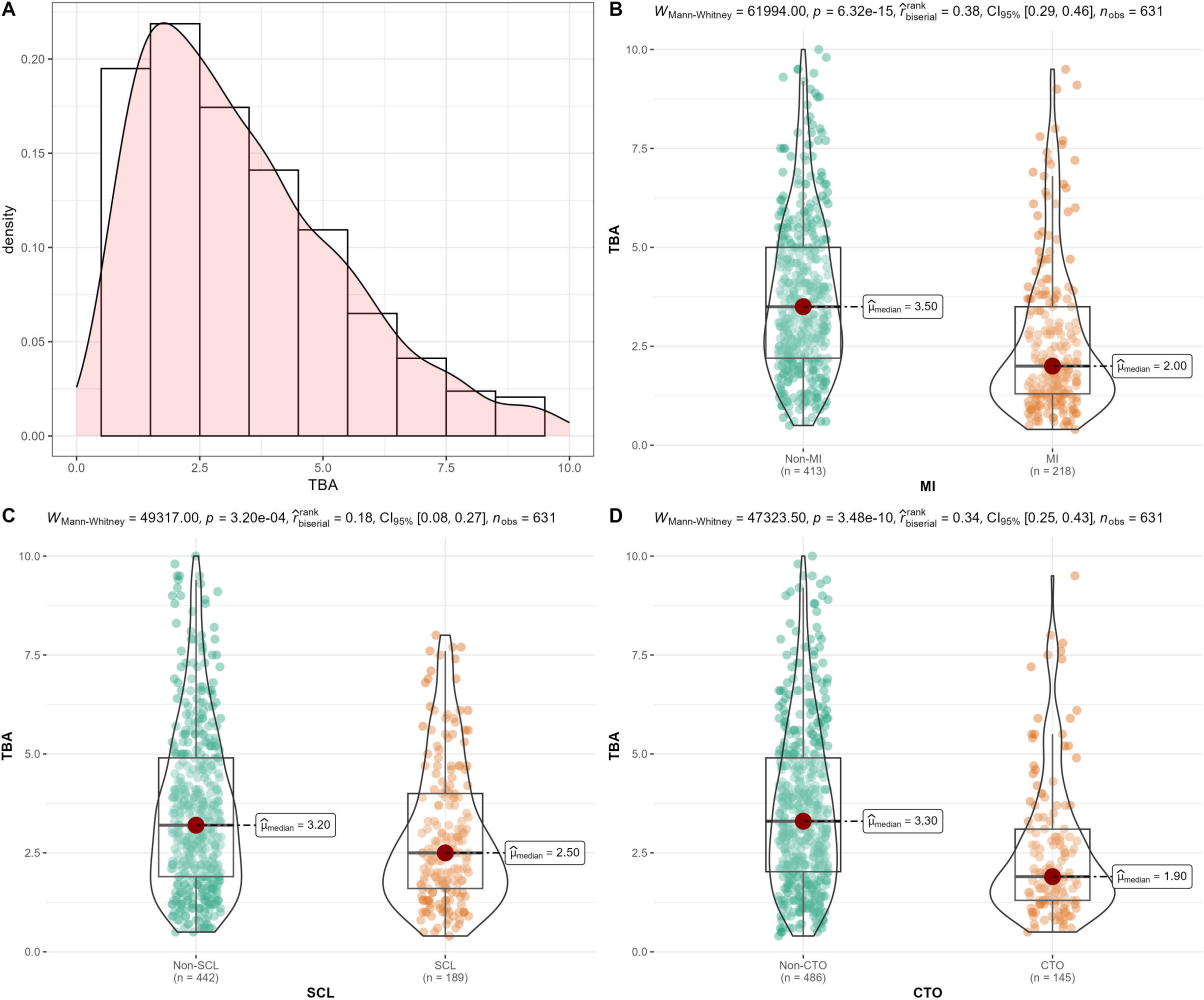
The population distribution of total bile acid (TBA). **(A)** TBA levels in total population; **(B)** TBA levels in premature coronary artery disease (PCAD) patients with and without first myocardial infarction (MI); **(C)** TBA levels in PCAD patients with and without severe coronary lesion (SCL); **(D)** TBA levels in PCAD patients with and without chronic total occlusion (CTO) lesions.

**Figure 1A** shows the distribution of TBA in the PCAD population, and it can be observed that TBA levels in the PCAD population are concentrated at 2-3 μmol/L. We found that PCAD patients with MI, SCL, and CTO lesions had significantly lower TBA levels than the corresponding groups (**Figures 1B–D**).

### 3.2 Correlation of TBA with inflammatory markers

**Figure 2:**
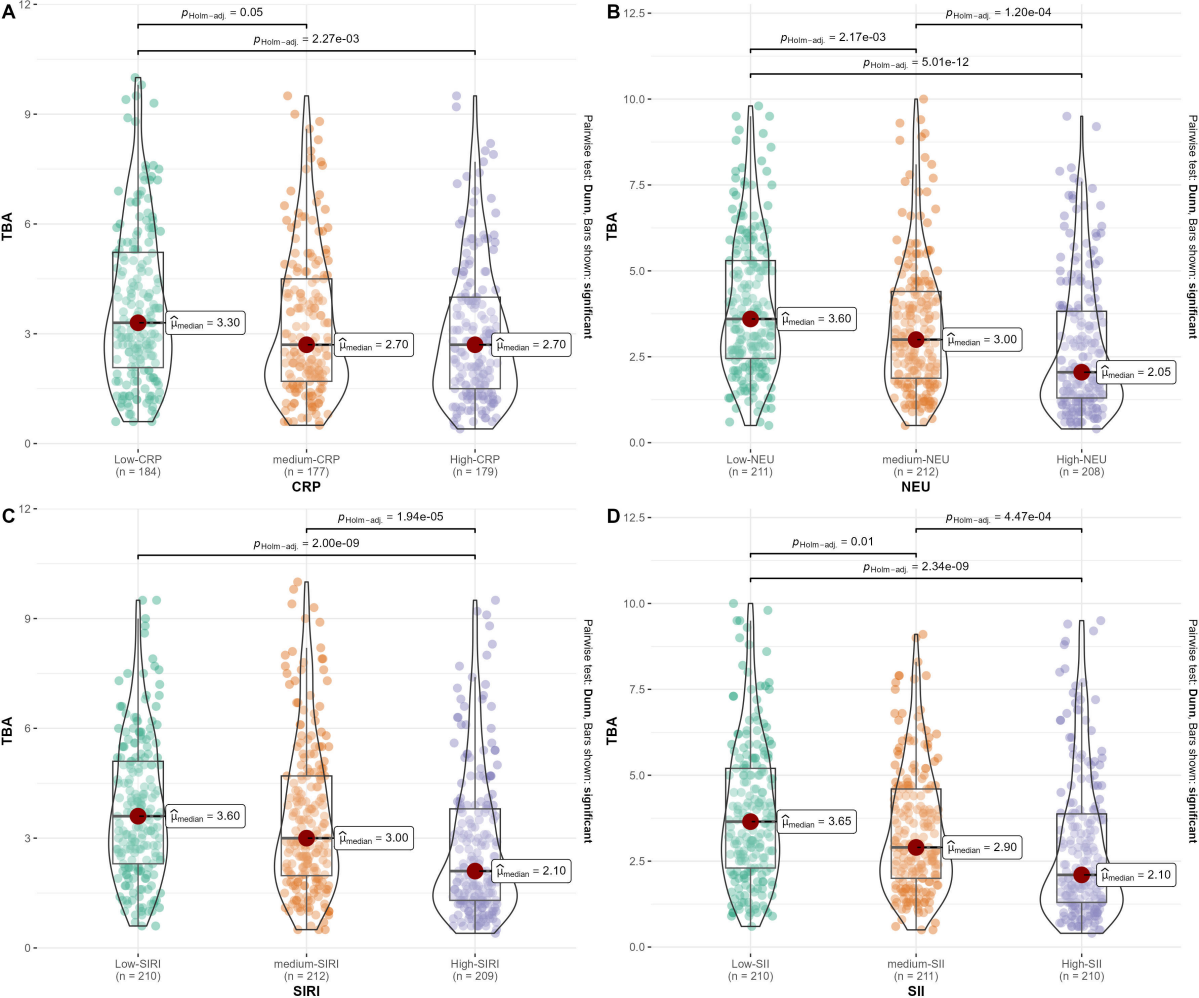
TBA levels in PCAD patients with different levels of inflammation. **(A)** TBA levels stratified by C-reactive protein (CRP); **(B)** TBA levels stratified by neutrophil (NEU); **(C)** TBA levels stratified by systemic inflammatory response index (SIRI); **(D)** TBA levels stratified by systemic immune-inflammation index (SII).

**Figure 3:**
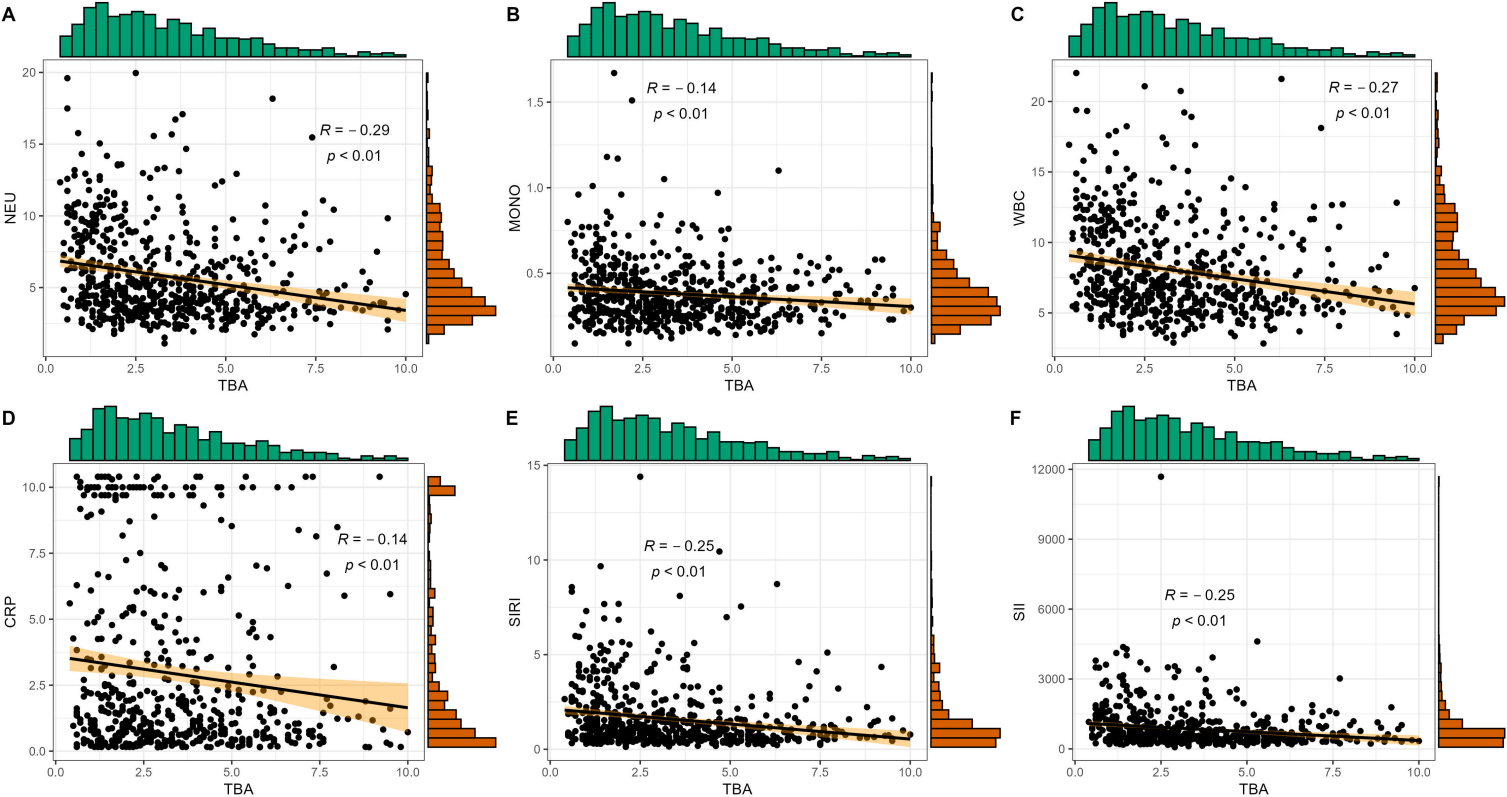
Correlations of TBA with inflammation indicators. **(A)** TBA and NEU; **(B)** TBA and monocytes (MONO); **(C)** TBA and white blood cell (WBC); **(D)** TBA and CRP; **(E)** TBA and SIRI; **(F)** TBA and SII.

As shown in **Figure 2**, we divided CRP, NEU, SIRI, and SII into three groups in tertiles to compare the serum TBA of PCAD patients with different levels of inflammation. We observed that the lower the level of CRP, NEU, SIRI, and SII, the higher the level of TBA in PCAD patients. In addition, as shown in **Figure 3**, Spearman’s correlation analysis also showed a significant negative correlation between NEU, Monocytes, WBC, CRP, SIRI, SII and TBA (ρNEU=-0.29, p<0.01; ρMonocytes=-0.14, p<0.01; ρWBC=-0.27, p<0.01; ρCRP=-0.14, p<0.01; ρSIRI=-0.25, p<0.01; ρSII=-0.25, p<0.01).

Both univariate and multivariate analyses showed that an increase in TBA was associated with a decrease in CRP, NEU, SIRI and SII in PCAD patients (**Table 2**). Univariate linear regression showed that for each unit increase in TBA, CRP, NEU, SIRI, and SII decreased by 0.19, 0.36, 0.16, and 76.01, respectively (CRP: β = -0.19, 95% CI: -0.33, -0.06; NEU: β = -0.36, 95% CI: -0.48, -0.24; SIRI: β = -0.16, 95% CI: -0.22, -0.10; SII: β = -76.01, 95% CI: -108.06, -43.96). When TBA was used as a categorical variable, patients with the highest TBA tertile had a decrease in CRP, NEU, SIRI, and SII of 1.09, 2.04, 0.92, and 417.46, respectively, compared with patients with the lowest TBA levels (CRP: β = -1.09, 95% CI: -1.76, -0.43; NEU: β = -2.04, 95% CI: -2.62, -1.47; SIRI: β = -0.92, 95% CI: -1.23, -0.62; SII: β = -417.46, 95% CI: -576.51, -258.41). The negative correlation was attenuated after adjusting for confounders, but remained statistically significant. Multivariate linear regression analysis showed a decrease of 0.13, 0.27, 0.12, and 55.25 in CRP, NEU, SIRI, and SII, respectively, for each unit increase in TBA (CRP: β = -0.13, 95% CI: -0.25, -0.02; NEU: β = -0.27, 95% CI: -0.37, -0.16; SIRI: β = -0.12, 95% CI: -0.17, -0.06; SII: β = -55.25, 95% CI: -85.75, -24.72).

**Table 2:** β (95% confidence interval) for the association of TBA with CRP, NEU, SIRI, and SII.

TBA	Unadjusted		Adjusted	
	β, (95% CI)	*p*	β, (95% CI)	*p*
CRP				
Continuous	-0.19 (-0.33, -0.06)	< 0.001	-0.13 (-0.25, -0.02)	0.025
Categorical				
Tertiles 1 (≤ 2.1)	Reference		Reference	
Tertiles 2 (2.1-3.9)	-0.70 (-1.37, -0.03)	0.040	-0.35 (-0.94, 0.23)	0.237
Tertiles 3 (>3.9)	-1.09 (-1.76, -0.43)	0.001	-0.59(-1.18, 0.00)	0.051
NEU				
Continuous	-0.36 (-0.48, -0.24)	< 0.001	-0.27 (-0.37, -0.16)	< 0.001
Categorical				
Tertiles 1 (≤ 2.1)	Reference		Reference	
Tertiles 2 (2.1-3.9)	-1.62 (-2.20, -1.04)	< 0.001	-1.06 (-1.58, -0.54)	< 0.001
Tertiles 3 (>3.9)	-2.04 (-2.62, -1.47)	< 0.001	-1.41 (-1.94, -0.88)	< 0.001
SIRI				
Continuous	-0.16 (-0.22, -0.10)	< 0.001	-0.12 (-0.17, -0.06)	< 0.001
Categorical				
Tertiles 1 (≤ 2.1)	Reference		Reference	
Tertiles 2 (2.1-3.9)	-0.75 (-1.06, -0.44)	< 0.001	-0.47 (-0.75, -0.19)	0.001
Tertiles 3 (>3.9)	-0.92 (-1.23, -0.62)	< 0.001	-0.62 (-0.90, -0.33)	< 0.001
SII				
Continuous	-76.01 (-108.06, -43.96)	< 0.001	-55.25 ( -85.78, -24.72)	< 0.001
Categorical				
Tertiles 1 (≤ 2.1)	Reference		Reference	
Tertiles 2 (2.1-3.9)	-297.48 (-457.12, -137.84)	< 0.001	-178.73 (-331.04, -26.41)	0.02
Tertiles 3 (>3.9)	-417.46 (-576.51, -258.41)	< 0.001	-280.08 (-434.91, -125.24)	< 0.001

**Figure 4:**
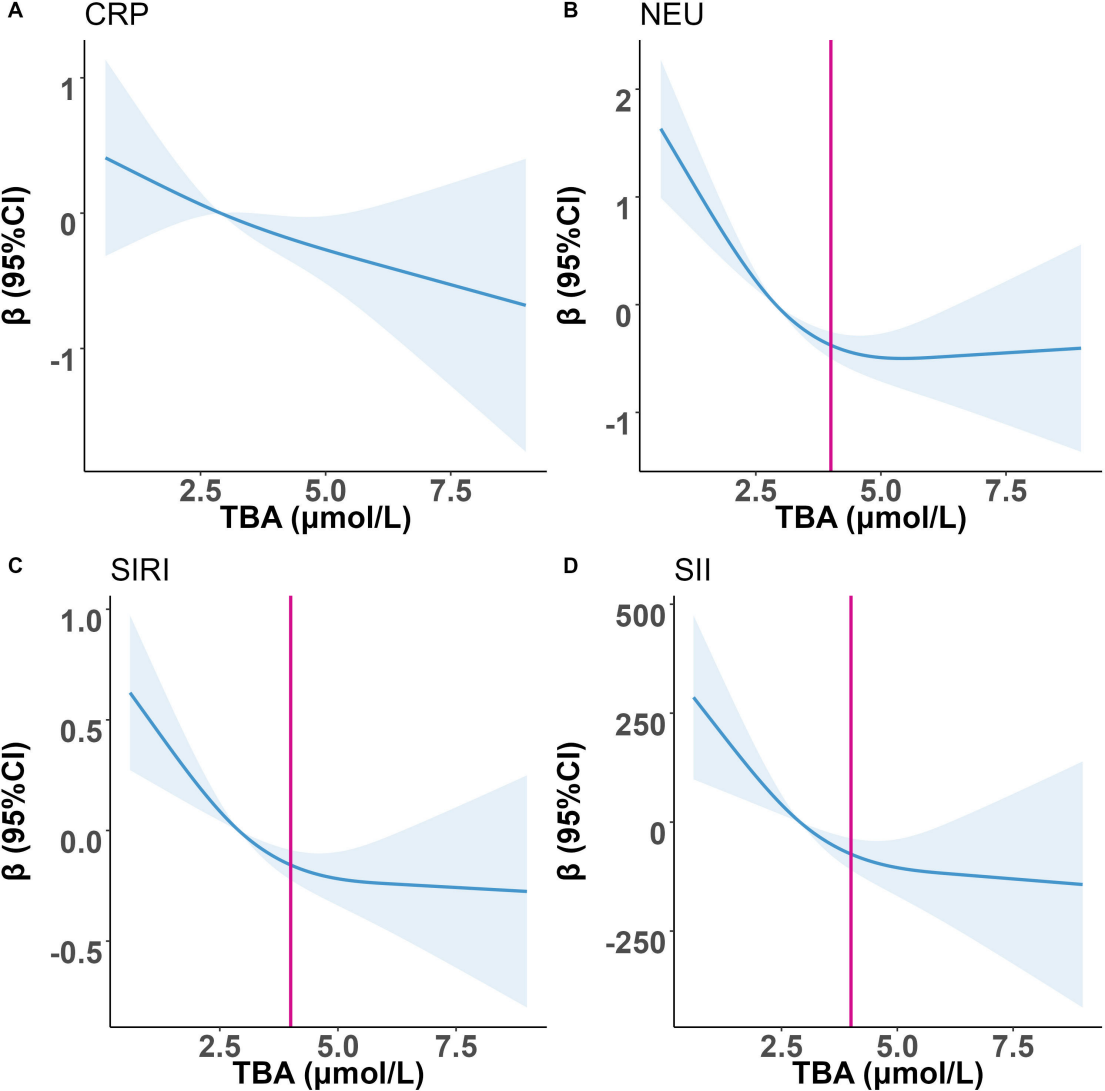
Restricted spline curve shows the relationship between TBA and inflammation indicators in the PCAD patients. **(A)** TBA and CRP; **(B)** TBA and NEU; **(C)** TBA and SIRI; **(D)** TBA and SII.

As shown in **Figure 4**, after adjusting for all covariates, we further visualized the association with CRP, NEU, SIRI, and SII using RCS curves when TBA was used as a continuous variable. As TBA increased, CRP, NEU, SIRI, and SII decreased in PCAD patients. We did not find a significant inflection point in the relationship between TBA and CRP. However, when the outcome variables were NEU, SIRI, and SII, the decreasing trend of the curve was significant when TBA was lower than 4 μmol/L, indicating that NEU, SIRI, and SII of PCAD patients decreased rapidly with the increase of TBA. When TBA exceeded 4 μmol/L, the downward trend of the curves gradually slowed down.

### 3.3 Correlation of TBA with first MI and the severity of coronary artery disease

Univariate and multivariate regression analyses revealed that TBA levels were negatively associated with the risk of MI, SCL, and CTO in PCAD patients (**Table 3**). In addition, we found that the higher the TBA level, the lower the Gensini score of PCAD patients (**Table 4**). Multivariate logistic regression analysis showed that each unit increase in TBA levels in PCAD patients was associated with a 26% (OR = 0.74, 95% CI: 0.66-0.82), 14% (OR = 0.86, 95% CI: 0.78-0.94), and 25% (OR = 0.75, 95% CI: 0.66-0.84) reduction in the risk of MI, SCL, and CTO, respectively.

**Table 3:** Odds ratio (95% confidence interval) for the association of TBA with MI, SCL, and CTO.

TBA	Unadjusted		Adjusted	
	OR, (95% CI)	*p*	OR, (95% CI)	*p*
MI				
Continuous	0.73 (0.66, 0.80)	<0.001	0.74 (0.66, 0.82)	<0.001
Categorical				
Tertiles 1 (≤ 2.1)	Reference		Reference	
Tertiles 2 (2.1-3.9)	0.31 (0.20, 0.46)	<0.001	0.33 (0.20, 0.53)	<0.001
Tertiles 3 (>3.9)	0.18 (0.11, 0.28)	<0.001	0.18 (0.11, 0.30)	<0.001
SCL				
Continuous	0.84 (0.77, 0.92)	<0.001	0.86 (0.78, 0.94)	<0.001
Categorical				
Tertiles 1 (≤ 2.1)	Reference		Reference	
Tertiles 2 (2.1-3.9)	0.63 (0.42, 0.94)	0.026	0.68 (0.44, 1.04)	0.078
Tertiles 3 (>3.9)	0.48 (0.31, 0.73)	<0.001	0.54 (0.34, 0.85)	0.008
CTO				
Continuous	0.73 (0.65, 0.81)	<0.001	0.75 (0.66, 0.84)	<0.001
Categorical				
Tertiles 1 (≤ 2.1)	Reference		Reference	
Tertiles 2 (2.1-3.9)	0.36 (0.23, 0.56)	<0.001	0.40 (0.24, 0.65)	<0.001
Tertiles 3 (>3.9)	0.21 (0.12, 0.34)	<0.001	0.23 (0.13, 0.39)	<0.001

**Table 4:** β (95% confidence interval) for the association of TBA with Gensini score.

TBA	Unadjusted		Adjusted	
	β, (95% CI)	*p*	β, (95% CI)	*p*
Continuous	-3.02 (-4.49, -1.56)	<0.001	-2.53 (-3.97, -1.10)	<0.001
Categorical				
Tertiles 1 (≤ 2.1)	Reference		Reference	
Tertiles 2 (2.1-3.9)	-13.24 (-20.54, -5.94)	<0.001	-11.55 (-18.70, -4.41)	0.002
Tertiles 3 (>3.9)	-17.02 (-24.29, -9.75)	<0.001	-13.79 (-21.05, -6.52)	<0.001

**Figure 5:**
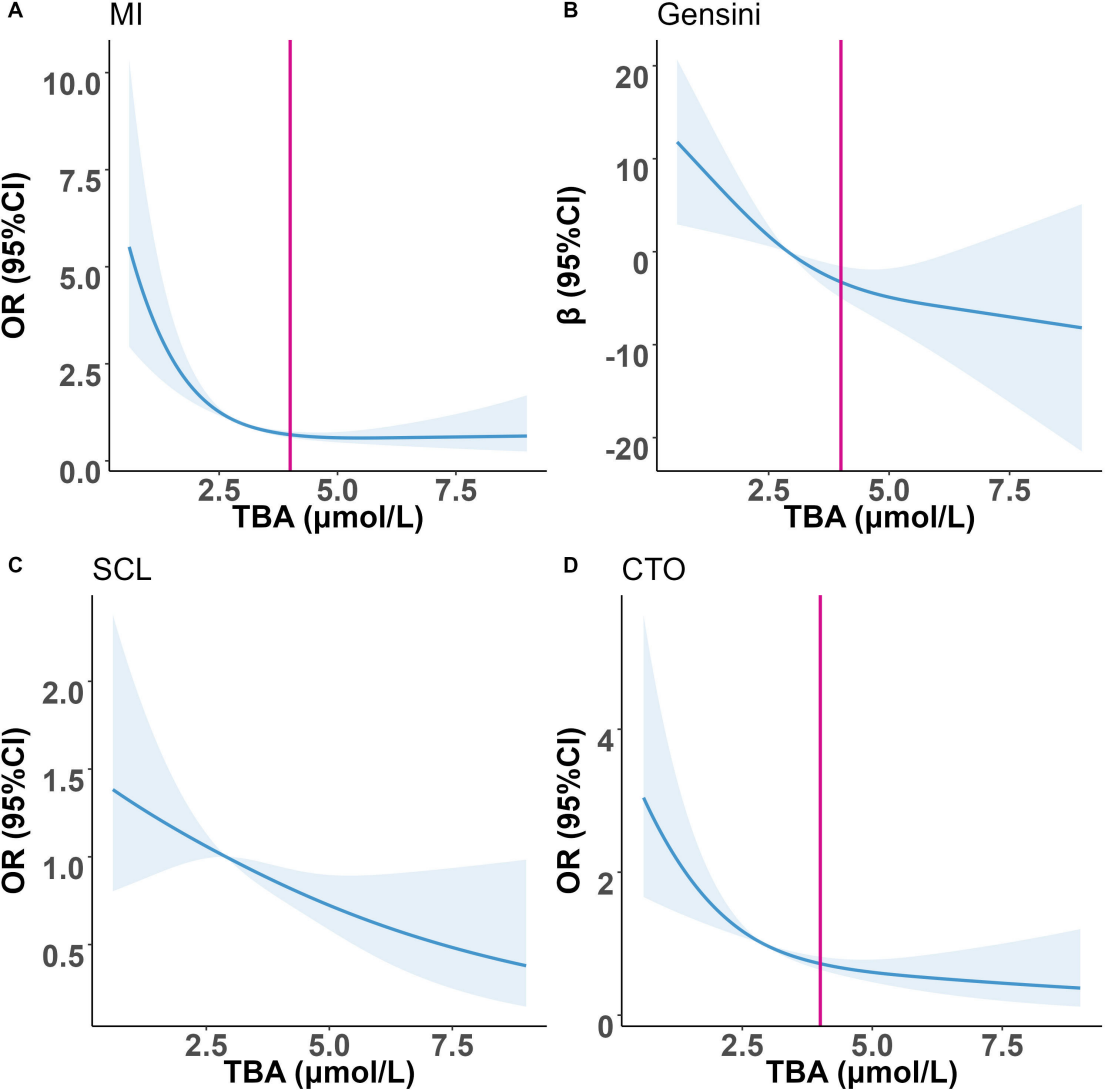
Restricted spline curve shows the relationship between TBA and outcomes in the PCAD patients. **(A)** TBA and MI; **(B)** TBA and Gensini; **(C)** TBA and SCL; **(D)** TBA and CTO.

The RCS curves suggested that the risk of first MI, SCL, CTO, and Gensini score in PCAD patients decreased with increasing TBA (**Figure 5**). We found that the association of TBA with first MI, CTO, and Gensini score showed a typical “L” shaped relationship. Based on the RCS curves, an inflection point of approximately 4 μmol/L was empirically identified, with a significant decreasing trend below this value and a plateau above it. It should be emphasized that that this inflection point should be viewed as an exploratory statistical finding from our dataset rather than a confirmed biological threshold. Nevertheless, the overall trend indicated that higher TBA concentrations were associated with reduced risks of first myocardial infarction and milder angiographic findings. We should pay attention to the PCAD group with lower TBA, which is associated with more severe coronary artery disease. However, we did not observe a significant inflection point in the RCS curves of TBA with SCL.

### 3.4 Mediating role of chronic inflammation

We performed multivariate analyses to assess the relationship between mediating variables (NEU, CRP, SIRI, and SII) and outcome variables (MI, CTO, SCL, and Gensini). As shown in **Supplementary Table 1**, we found significant positive associations between almost all inflammatory markers and outcome variables in PCAD patients. However, this study did not find significant associations between CRP, SIRI, SII, and SCL. Therefore, CRP, SIRI, and SII did not serve as mediating variables between TBA and SCL.

**Figure 6:**
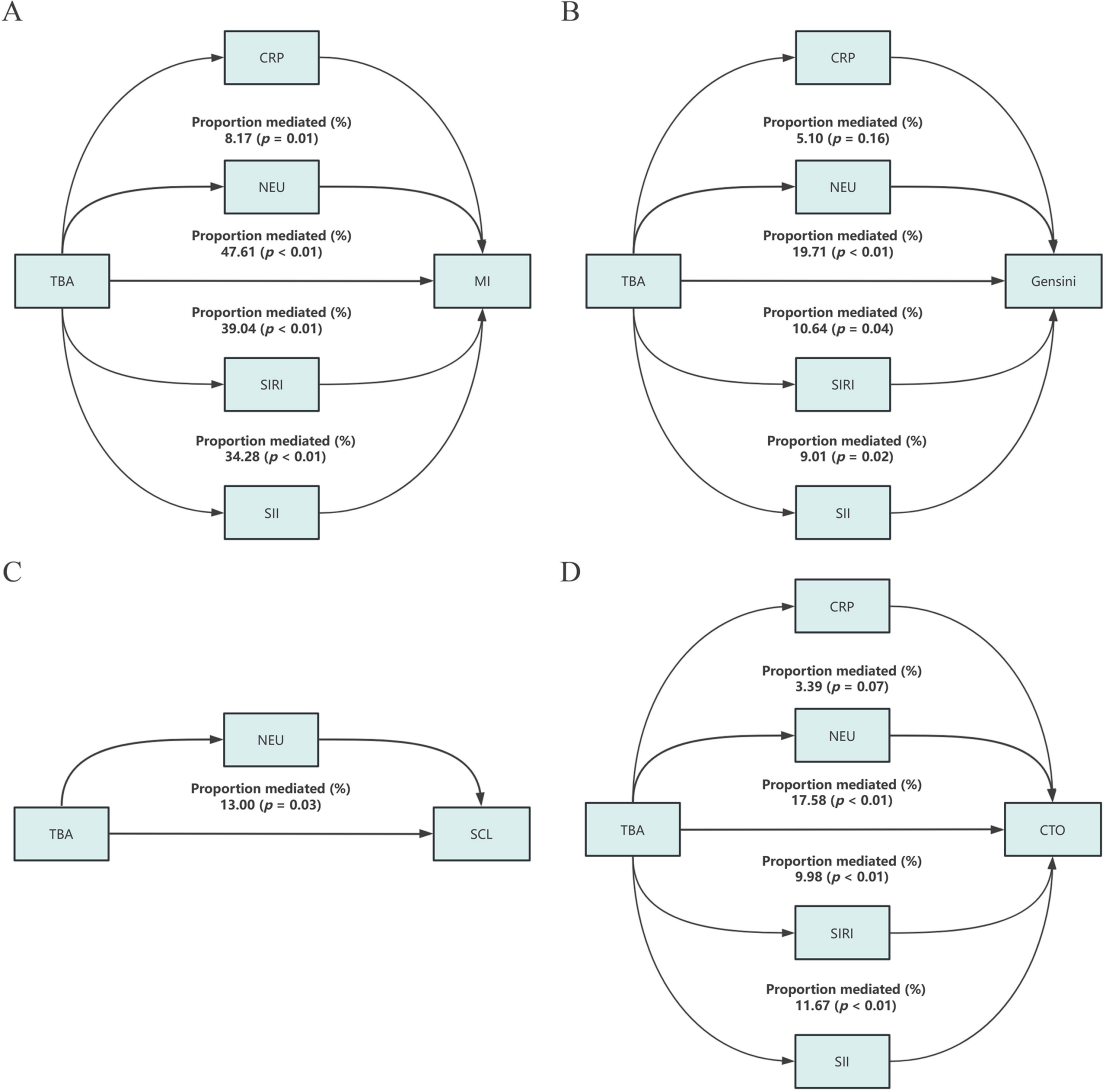
Mediating role of chronic inflammatory indicators in the relationship between TBA and different outcomes in PCAD Patients. **(A)** TBA and MI; **(B)** TBA and Gensini; **(C)** TBA and SCL; **(D)** TBA and CTO. Each mediator was analyzed in separate single-mediator models, and the proportions mediated are not additive due to correlations/overlapping information among the mediators.

**Figure 6** displays the mediation analysis examining the role of inflammation in the association between TBA and outcome variables. **Supplementary Tables 2**, **3** show the coefficients for total and indirect effects in the mediated effects analysis. NEU mediated 47.61%, 13.00%, 17.58% and 19.71% of the associations of TBA with MI, SCL, CTO, and Gensini scores, respectively. SIRI mediated 39.04%, 9.98%, and 10.64% of the associations of TBA with MI, CTO, and Gensini score, respectively. SII mediated 34.28%, 11.67%, and 9.01% of the associations of TBA with MI, CTO, and Gensini score, respectively. CRP mediated only 8.17% of the TBA-MI association. However, we did not find a mediating effect of CRP for TBA with the Gensini score and CTO.

## 4 Discussion

In this study, we evaluated the relationship between TBA and the degree of first MI and coronary artery disease in PCAD patients for the first time. We found that lower TBA levels within the normal range were strongly associated with an increased risk of first MI in these patients. The lower the TBA, the more severe the degree of coronary artery disease in PCAD patients. We also report a significant negative correlation between TBA and levels of inflammatory markers. More importantly, mediation analysis indicated that the inverse association between TBA levels and the severity of coronary lesions in PCAD patients was partially mediated by inflammatory markers, suggesting a potential anti-inflammatory pathway.

As early as the 1980s, H Simonen et al. identified abnormalities in bile acid synthesis in patients with familial hypercholesterolemia (20). Subsequently, Gideon Charach et al. also found significantly lower levels of bile acid excretion in CAD patients compared with non-CAD patients (21). Their team reconfirmed the lower bile acid excretion rate in CAD patients after a long-term follow-up (7). Although the studies had small sample sizes and focused only on fecal bile acids, they suggested that bile acids may play a protective role in the development of CAD. In contrast, Carine Steiner et al. did not find a significant association between bile acids and CAD when they compared data from 75 CAD patients and 75 non-CAD controls (22). However, a subsequent study overturned the findings of Carine Steiner et al. after analyzing 7438 patients admitted to the hospital for chest pain (9). It found that patients with lower TBA levels had a higher risk of being diagnosed with CAD and developing MI than patients with higher TBA levels. These patients also had more severe CAD (9). In addition, TBA reduces the incidence of adverse cardiovascular events in patients with acute coronary syndromes or CTO (23, 24). A recent study demonstrated a partial mediating role of TBA between Lactobacillus intestinalis and prognosis in patients with acute coronary syndromes (10). To date, no studies have examined TBA in the PCAD population. TBA is associated with a lower risk of first MI and less severe coronary artery disease in PCAD patients. Most cases of acute MIs are caused by acute occlusion of the lumen by thrombosis from rupture of coronary atherosclerotic plaques. However, no clinical study has yet explored the correlation between TBA and plaque components (e.g., fibrous cap thickness and lipid core ratio), and thus the mechanism underlying this association is unclear.

Previous studies have shown that inflammatory markers, endothelial dysfunction, and oxidative stress play an important role in the development of atherosclerosis. Classical inflammatory markers include NEU, NLR, CRP, IL-6, and soluble adhesion molecules. The chronic inflammatory state in the body accelerates the progression of atherosclerosis and is associated with a range of adverse cardiovascular events. In our study, increased inflammatory markers were also associated with a more severe degree of coronary artery disease in PCAD patients. Moreover, the innate and adaptive immune systems play a significant role in the development and progression of atherosclerosis (25). Therefore, we hypothesized that the protective association of TBA in PCAD patients may be linked to its potential anti-inflammatory and immunomodulatory effects.

Previous basic studies have suggested that TBA inhibits the activation of inflammatory pathways. TBA activates the TGR5 and increases cAMP production in macrophages, thereby inhibiting lipopolysaccharide-induced release of inflammatory cytokines and cellular phagocytic activity (26, 27). Inhibition of inflammation by TGR5 activation may play a beneficial role in halting the progression of atherosclerosis. However, it is worth noting that the accumulation of bile acids in the liver due to cholestasis instead promotes the inflammatory response of the body (28). Higher TBA may indicate underlying liver injury or biliary obstruction, which may adversely affect the heart. High TBA levels in cirrhotic patients have been reported to be associated with cardiac insufficiency (29). TBA can be elevated up to 100 times the normal value in patients with cirrhosis combined with cardiomyopathy (29). Therefore, we excluded subjects with a TBA above 10 µmol/L based on clinical reference values and explored only the effect of bile acid levels within the normal range on outcome variables. Our study demonstrated that PCAD patients with lower TBA levels had higher levels of inflammation, particularly NEU, which was associated with the extent of coronary lesions. Mediation effect analysis confirmed our conjecture that inflammatory indicators represented by NEU play a key role in the association of bile acids with first MI and coronary lesions in PCAD.

Although our study suggests that TBA is beneficial for patients with PCAD, we need to emphasize that the complexity of bile acids composition. Multiple bile acid components can play different, or even opposite, roles in the pathophysiology of CAD. For example, a prospective study of 1234 patients with new-onset type 2 diabetes mellitus using the Dongfeng-Tongji cohort found a linear correlation between higher concentrations of free secondary bile acids (especially deoxycholic acid) and a higher risk of cardiovascular diseases (30). Chenodeoxycholic acid, deoxycholic acid, and lithocholic acid have also been linked to endothelial dysfunction and vascular injury formation (31). However, ursodeoxycholic Acid (UDCA) has shown protective effects on the cardiovascular system. UDCA promotes the lysis of cholesterol crystals in macrophages and reduces the production of IL-1β (32). In ApoE knockout rats, UDCA inhibited the formation of atherosclerotic plaques and regressed the formed coronary lesions (32). TBA is the sum of all circulating bile acids. Therefore, we cannot speculate on the role of specific components of TBA in PCAD patients. We consider it necessary to use metabolomics to explore the roles of different bile acid components in PCAD patients.

Despite the innovative nature of this study, there are some limitations to our study. First, the cross-sectional study design prevents establishing causality between TBA and outcome variables. It is unclear whether bile acids actively modulate inflammatory responses or simply reflect metabolic status associated with disease severity. Future studies using functional experiments—such as treating immune cells with physiologically relevant bile acid mixtures and measuring inflammatory cytokine production—are needed to establish causality. Second, as a single-center study of only young Chinese PCAD patients, generalizability is limited. Third, because participants were not new cases, but hospital patients, Berkson bias was unavoidable. Fourth, we did not measure specific bile acids components, so their roles require further investigation. Fifth, the mediation analysis should be viewed as a statistical test of mediation or an indirect association, not as proof of causal biological pathways. Causal inferences require strong assumptions, including correct temporal ordering between exposure, mediator, and outcome, as well as no unmeasured confounding of the exposure-mediator, mediator-outcome, and exposure-outcome relationships. Given the cross-sectional nature of our data and the retrospective observational design, these assumptions are difficult to verify. Future prospective studies are needed to confirm temporal relationships and potential underlying mechanisms. Sixth, dietary effects on bile acid metabolism may confound associations. However, clinical dietary assessment is is challenging, and residual confounding cannot be excluded. Seventh, non-fasting status and acute-phase physiology—particularly in MI patients—may affect both TBA and inflammatory indices, which can complicate the interpretation of their associations with MI. Some MI patients requiring emergency PCI are sampled at admission (often non-fasting). Postprandial status typically elevates TBA, but our study found significantly lower TBA levels in MI patients. This discrepancy suggests that the observed TBA reduction is unlikely driven by non-fasting status and may reflect true pathophysiological changes. Nevertheless, acute-phase responses could still influence inflammation and affect mediation estimates. Thus, mediation findings should be interpreted with caution. Eighth, while the RCS curves revealed a nonlinear relationship, the inflection point of approximately 4 μmol/L lacks formal validation. Future studies should employ external cohorts to confirm whether this represents a stable biological transition.

Additionally, our study is limited by its reliance on a single routine measurement of TBA. While the statistical associations are robust, blood contains a complex milieu of circulating biomarkers—including metabolites, proteins, and cell-free DNA (cfDNA)—that collectively inform disease status. Emerging research in liquid biopsy, particularly in oncology, has demonstrated that cfDNA methylation and fragmentomic signatures can capture systemic pathological changes (33). To enhance the translational relevance of TBA as a cardiovascular biomarker, future investigations should adopt a multi-modal approach. Integrating metabolic markers with lipidomic profiles or cfDNA-based signals could provide orthogonal validation and reveal whether TBA reflects broader systemic processes. Such integration would position bile acid analysis within the evolving paradigm of multi-analyte liquid biopsies for cardiovascular risk assessment.

## 5 Conclusion

In conclusion, our study revealed that TBA was associated with a decreased risk of first MI and a reduced degree of coronary stenosis in PCAD patients. The higher the TBA, the lower the level of chronic inflammation in PCAD patients. More importantly, the statistical mediation analysis revealed that inflammatory indices statistically accounted for the association between TBA and reduced coronary lesion severity.

## Data Availability

The raw data supporting the conclusions of this article will be made available by the authors, without undue reservation.
