# Supplementary material for: Serum total bile acids within the normal range are inversely associated with inflammatory indices and Gensini score in patients with premature coronary artery disease
Source: Front Immunol. 2026 Apr 16;17:1790698. doi: 10.3389/fimmu.2026.1790698 (PMC13128411; doi:10.3389/fimmu.2026.1790698)
Supplement: Supplementary file 1 [file DataSheet1.docx]

Supplementary Material

# Supplementary Tables

| **Table S1. The association of mediating variables (NEU, CRP, SIRI, SII) with outcome variables (MI, CTO, SCL, Gensini).** | | | | | | | | |  |
| --- | --- | --- | --- | --- | --- | --- | --- | --- | --- |
|  | | MI | | SCL | | CTO | | Gensini | |
|  |  | OR, (95% CI) | *p* | OR, (95% CI) | *p* | OR, (95% CI) | *p* | β,(95% CI) | *p* |
| NEU | | 2.09 (1.84, 2.41) | <0.001 | 1.10 (1.03, 1.17) | 0.004 | 1.24 (1.16, 1.34) | <0.001 | 2.17 (1.11, 3.23) | <0.001 |
| CRP | | 1.25 (1.16, 1.36) | <0.001 | 1.05 (0.99, 1.13) | 0.110 | 1.10 (1.02, 1.18) | 0.009 | 1.24 (0.07, 2.42) | 0.039 |
| SIRI | | 3.87 (2.93, 5.26) | <0.001 | 1.11 (0.99, 1.25) | 0.065 | 1.34 (1.19, 1.53) | <0.001 | 2.91 (0.92, 4.89) | 0.004 |
| SII (per 100 units) | | 1.28 (1.22, 1.36) | <0.001 | 1.02 (1.00,1.05) | 0.063 | 1.07 (1.04, 1.10) | <0.001 | 0.51 (0.14, 0.88) | 0.007 |

ssss

| **Table S2. The Indirect effect for the mediating role of inflammation in the association between TBA and outcome variables (MI, CTO, SCL, Gensini).** | | | | |
| --- | --- | --- | --- | --- |
|  | MI | CTO | SCL | Gensini |
| TBA→CRP | -0.005(-0.010, -0.001) | -0.002(-0.005, 0.000) | -0.001(-0.004, 0.001) | -0.142(-0.431, 0.050) |
| TBA→NEU | -0.023(-0.032, -0.014) | -0.009(-0.015, -0.004) | -0.004(-0.009, -0.001) | -0.503(-0.955, -0.141) |
| TBA→SIRI | -0.018(-0.027, -0.010) | -0.005(-0.010, -0.002) | -0.002(-0.005, 0.001) | -0.279(-0.617, -0.014) |
| TBA→SII | -0.016(-0.024, -0.009) | -0.006(-0.010, -0.002) | -0.002(-0.005, 0.001) | -0.233(-0.484, -0.033) |

| **Table S3. The total effect for the mediating role of inflammation in the association between TBA and outcome variables (MI, CTO, SCL, Gensini).** | | | | |
| --- | --- | --- | --- | --- |
|  | MI | CTO | SCL | Gensini |
| TBA→CRP | -0.059(-0.080, -0.037) | -0.055(-0.085, -0.026) | -0.030(-0.054, -0.008) | -2.505(-4.038, -0.981) |
| TBA→NEU | -0.048(-0.068, -0.028) | -0.051(-0.079, -0.026) | -0.032(-0.054, -0.011) | -2.527(-3.872, -1.191) |
| TBA→SIRI | -0.047(-0.065, -0.028) | -0.052(-0.081, -0.026) | -0.032(-0.054, -0.011) | -2.527(-3.869, -1.182) |
| TBA→SII | -0.047(-0.064, -0.029) | -0.051(-0.079, -0.025) | -0.030(-0.054, -0.011) | -2.525(-3.877, -1.182) |
